# Arterial spin labeling (ASL-MRI) versus fluorodeoxyglucose-PET (FDG-PET) in diagnosing dementia: a systematic review and meta-analysis

**DOI:** 10.1186/s12883-023-03432-y

**Published:** 2023-10-24

**Authors:** Hiba Haidar, Rania El Majzoub, Shorouk Hajeer, Linda Abou Abbas

**Affiliations:** 1 Neuroscience Research Center, Faculty of Medical Sciences, Lebanese University, Beirut, Lebanon; 2 Department of Biomedical Sciences, School of Pharmacy, Lebanese International University, Beirut, Lebanon; 3 Laboratory of Cancer Biology and Molecular Immunology, Faculty of Sciences-I, Lebanese University, Beirut, Lebanon

**Keywords:** Dementia, FDG-PET, ASL-MRI, Diagnostic accuracy, Review, Meta-analysis

## Abstract

**Background:**

Dementia is generally caused by neurodegenerative diseases affecting the brain, which leads to a progressive neurocognitive decline characterized by inability to perform major higher functioning tasks. Fluorodeoxyglucose-positron emission tomography (FDG-PET) scan is one of the main imaging tests performed for diagnostic purposes. However, with FDG-PET being quite expensive and not widely available, an attempt to find an alternative is set. Arterial-spin-labelling magnetic resonance imaging (ASL-MRI) is an increasingly investigated substitute to FDG-PET for the diagnosis of dementia. Thereby, the main purpose of this systematic review and meta-analysis is to compare the diagnostic ability of FDG-PET and ASL-MRI in detecting dementia.

**Methods:**

PRISMA checklist for diagnostic test accuracy was employed in outlining this paper. A literature search was done using several search engines including PubMed, Core, and Cochrane. Two researchers (HH and SH) extracted the essential information from all included articles. Risk of bias was evaluated by the Quality Assessment of Diagnostic Accuracy Studies tool, version 2 (QUADAS-2). A qualitative analysis and summary of studies’ results were provided. In addition, a meta-analysis was executed based on the studies which involved sensitivity and specificity measures of diagnostic accuracy.

**Results:**

Fourteen total studies were included in the given review. Qualitative analysis of the articles showed that nine studies demonstrated an overlap between metabolic and perfused brain maps as derived by FDG-PET and ASL-MRI respectively, while the remaining five studies registered significant differences across both modalities, with superiority to FDG-PET. As for the meta-analysis implemented, summary ROC-curve analysis revealed that FDG-PET performed better than ASL-MRI, with pooled sensitivity being significantly higher for FDG-PET.

**Conclusions:**

Comparing the diagnostic value of FDG-PET and ASL-MRI, the results of this systematic review and meta-analysis indicate that FDG-PET still has an advantage over ASL-MRI. Such implication could be related to the technical differences relating to both modalities, with ASL-MRI having lower temporal resolution. It’s worth mentioning that specificity was rather quite similar among both modalities and some studies found an overridden metabolic and perfused images. These findings call for future research to focus their scope of investigation while exploring the diagnostic value of ASL-MRI.

## Background

Dementia is derived from the Latin word “de mens” which describes the deteriorating aspect of mental capabilities [1]. According to the World Health Organization (WHO), dementia is defined as a health problem resulting from a chronic disease inflicting the brain [2]. The result of this progressive decline is the inability of a person to perform some major higher cortical functions including memory, learning, and thinking [3]. Neuropsychiatric symptoms are commonly found in demented patients, and they involve depression, agitation, and apathy [4]. Worldwide, it is estimated that 44 million people are suffering from dementia, with that number doubling every 20 years till the year 2050 [5]. Each year there are around 7.7 million new cases of dementia registered with most of these cases centered in low and middle-income countries [6].

Alzheimer’s disease (AD) is the most common neurodegenerative disease affected by and it constitutes 75% of all dementia cases [7]. Alzheimer’s disease is characterized by memory loss and language-related problems [8]. Vascular dementia (VD) is the second cause contributing to dementia cases [2]. It originates from an arterial disease compromising the blood supply to the brain leading to neuronal damage [9]. Other causes include dementia with Lewy bodies (DLB), explaining 10% of dementia cases [10].

In an attempt to diagnose patients with dementia, a number of clinical and cognitive tests are prone to take place to confirm the designated disease. The earlier the diagnosis is made, the better the prognosis [11]. Neuroimaging is an important subset of these diagnostic tests. They offer biomarkers, which are considered helpful predictors in following up the trajectory of the disease [12]. Neuroimaging techniques employed include structural imaging, like computed tomography (CT), and functional imaging. The latter involves mainly positron emission tomography (PET) and functional magnetic resonance imaging (fMRI) [13].

Fluorodeoxyglucose-PET (FDG-PET) scan is employed in the early assessment and differential diagnosis of dementia [14]. Neuroimaging biomarkers detected on FDG-PET include hypometabolism patterns in the posterior cingulate gyrus, parietal lobe, frontal lobe, and anterior and posterior temporal lobes, with sensorimotor cortex involvement [15]. Despite FDG-PET being highly beneficial in the diagnosis of dementia, still several shortcomings are facing its easy maneuver which involve high exposure to radiation, inaccessibility in many developing countries, and its high cost [16]. Thus, other substitutes were investigated to replace the usage of FDG-PET in detecting dementia including arterial-spin-labelling magnetic resonance imaging (ASL-MRI). This technique in MRI uses a labelled magnetic arterial tracer to measure regional cerebral blood flow [17]; as glucose metabolism and cerebral blood flow are biologically coupled [18].

Several studies in the literature have explored and compared the ability of FDG-PET and ASL-MRI to detect dementia. Ceccarini et al. compared the diagnostic ability of ASL-MRI and FDG-PET in differentiating dementia patients and controls [19]. Equivalent specificity was registered (0.7) with higher sensitivity for FDG-PET (0.93). In a case-control study simultaneously comparing ASL-MRI and FDG-PET diagnostic values in a sample of Alzheimer’s disease and frontotemporal dementia patients, Fällmar et al. results have shown higher specificity for ASL-MRI (0.84), but lower sensitivity in comparison to FDG-PET (0.53 versus 0.96) [20].

Henceforth, the former findings derived from literature suggest that FDG-PET and ASL-MRI are comparable, and that both can offer important information about the diagnosis of demented patients. With that kept in mind, our main purpose in this systematic review and meta-analysis is to summarize the results relating to the diagnostic values of ASL-MRI and FDG-PET in correctly distinguishing dementia cases. Our review will combine a qualitative assessment of all studies, and a meta-analysis summarizing sensitivity and specificity measures.

## Materials and methods

The following systematic review follows the guidelines of Preferred Reporting Items for Systematic Reviews and Meta-Analyses- Diagnostic Test Accuracy (PRISMA-DTA) [21] for reporting systematic reviews of diagnostic test accuracy studies.

### Eligibility criteria

Studies were considered eligible for recruitment if they involved dementia patients of any disease, where these patients were examined using both ASL-MRI and FDG-PET during the same interval of time. There was no restriction on time nor gender. Studies were excluded if recruited patients in the retrieved studies were younger than 18 years of age, had an accompanying history of diseases like epilepsy, have received corticosteroid treatment, or underwent radiotherapy or other treatments before imaging studies were attained.

### Information sources

An electronic database search was employed to extract the eligible articles from literature. Electronic databases searched included PubMed, CORE, Cochrane, and additional academic journals being biomedical central journal, Neurology journal, Journal of Neuroimaging, Radiology Journal, Annals of Neurology, and Journal of MRI.

### Search strategy

Literature was searched through online electronic databases, from March till May 2022, using MeSH terms that involved “dementia”, “FDG-PET”, “PET-scan”, “ASL-MRI”, and “arterial spin labeling”. The former MeSH terms were combined by Boolean operators “OR” and “AND”. The adjusted search overall was in the following form: (“dementia” AND (“FDG-PET” OR “PET-scan”) AND (“Arterial spin labeling” OR “ASL”)).

### Study selection

Two reviewers (HH and SH) have separately and independently navigated the literature and assessed the articles for inclusion. If there was any disagreement among the reviewers, a discussion was set to resolve it.

### Data extraction and quality assessment

Information extracted from all included articles is as follows: study’s citation, study design, target condition, sample size of healthy and diseased patients, index tests used, assessment method for analyzing the data received from the employed imaging modalities (visual versus quantitative), the diagnostic accuracy measures reported, and the main findings of the study.

In addition to the previous process of data extraction, studies that provided diagnostic accuracy measures in the form of sensitivity and specificity had an additional separate process of data extraction. Data extracted or derived from these latter articles involved the study’s citation, target condition, assessment method of data acquired from index tests, sensitivity, specificity, positive predictive value, and negative predictive value.

Studies’ risk of bias was evaluated by the Quality Assessment of Diagnostic Accuracy Studies tool, version 2 (QUADAS-2) [22].

### Data interpretation and statistical analysis

Eligible articles were qualitatively assessed and interpreted. Imaging-based findings were separately and qualitatively summarized for all articles. On the other side, studies that reported sensitivity and specificity measures were part of the meta-analysis executed.

Qualitative analysis of the articles consisted mainly of providing a narrative summary of the findings related to metabolized versus perfused brain patterns, while categorizing them based on the nature of the dementia-related disease.

As for the meta-analysis, sensitivity and specificity diagnostic measures were extracted from selected studies and converted into true positive (TP), true negative (TN), false positive (FP), and false negative (FN) values to be able to undertake the necessary statistical analyses. Pooled sensitivity and specificity values were estimated and compared while being clearly demonstrated using Forest plots per imaging modality.

A summary receiver-operator characteristic curve (SROC-curve) was constructed based on a bivariate model provided by Reitsma [23]. The applied approach considers the correlation between sensitivity and specificity and within-study variations. The necessary parameters required for fitting the model into the SROC curve were extracted using MetaDTA [24] online software. Area under the curve (AUC) and heterogeneity index I2 were calculated with the statistical analysis software RStudio using mada package.

All statistical analyses, summary curves and forest plots were performed using MetaDTA [24], RStudio version 4.2.1, and review manager (REVMAN) version 5.4.1.

## Results

### Literature search findings

**Fig. 1:**
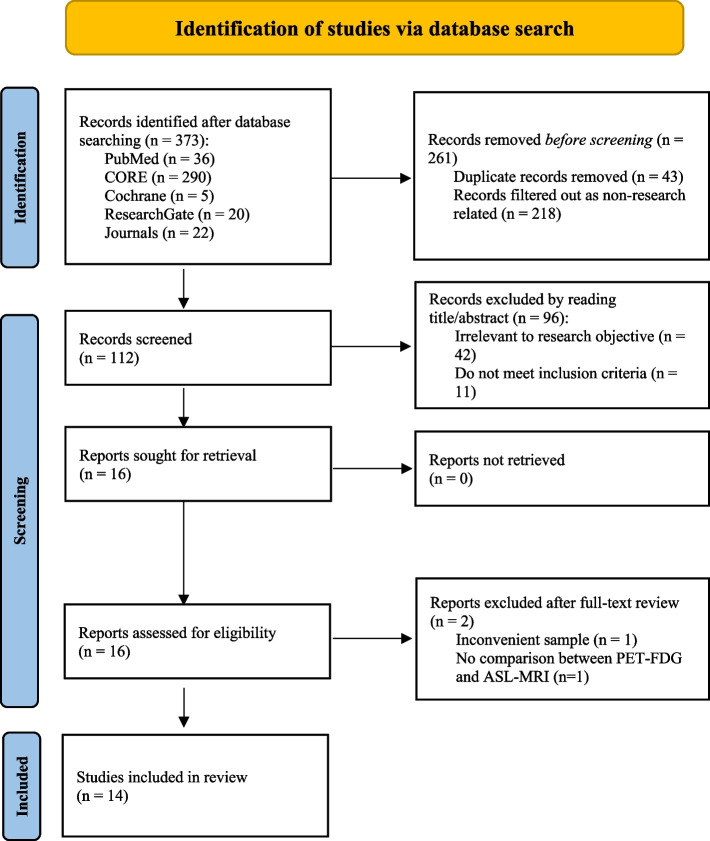
PRISMA flow diagram used for studies’ selection

The overall literature search yielded a total of 373 articles, of which 112 articles were screened for eligibility after reading through their title and abstract only. Of these 112 articles, only 16 articles were sought for retrieval. Fourteen articles were overall included (Fig. 1).

### Study characteristics

All articles included are case-control studies except for two [25, 26]. All of these studies have incorporated FDG-PET and ASL-MRI to compare their diagnostic value in identifying dementia. Data retrieved from both modalities were blindly compared and interpreted using visual assessment (performed by neuroradiologists and imaging specialists), quantitative assessment which involved statistical models for comparison, or both methods.

For comparison purposes between both imaging techniques, varying diagnostic accuracy measures were thereby documented including receiver-operator characteristic curve analyses, sensitivity, and specificity. Nonetheless, imaging-derived brain maps were compared using several analysis techniques like voxel-wise, region-of-interest, and volume-of-interest analyses (Table 1).

**Table 1:** Study characteristics of included articles

Citation	Study Design	Target Condition	Sample Size (Diseased/Healthy)	Index Tests	Assessment Method (Visual/Quantitative)	Diagnostic Accuracy Measures	Findings
Anazodo et al. [2017] [27]	Case-control	Frontotemporal dementia (FTD)	10/10	ASL-MRI /FDG-PET	Visual	Sensitivity/specificity	ASL showed lower sensitivity (0.6667), specificity (0.6212), and inter-rater reliability (0.2) than FDG-PET (being 0.8843/0.9091/0.61 respectively)
					Quantitative	t-score maps	Hypometabolism areas exceeded that of hypoperfused ones
Ceccarini et al. [2020] [19]	Case-control	Different types of dementia	27/30	Enhanced multiplane tagging ASL-MRI/ FDG-PET	Visual	Sensitivity, specificity, and interobserver agreement	Similar specificity (0.7) was registered among both modalities, but higher sensitivity (0.93) and interobserver agreement (0.64) for FDG-PET
					Quantitative	Volume of interest (VOI)–based analysis/z-scores	FDG-PET revealed more volume and intensity abnormalities than ASL-MRI. FDG-PET-based VOI reported higher z-scores with varying brain regions
Chen et al. [2011] [28]	Case-control	Alzheimer’s disease (AD)	15/19	Pseudo continuous ASL-MRI /FDG-PET	Quantitative	Statistical parametric mapping and regions of interest (ROI) analysis	Perfusion and metabolism maps showed an overlap in various brain regions including bilateral angular gyri and posterior cingulate ROI results revealed similar abnormalities in diseased and healthy patients
						Linear correlation maps of perfusion and metabolism to neuropsychological test scores	Both modalities were able to correctly discriminate neural networks related to psychological tests. Positive correlations were observed in the dorsolateral prefrontal cortex and bilateral inferior parietal lobes
Corouge et al. [2012] [29]	Case-control	Semantic dementia (SD)	6/9	Pulsed ASL-MRI /FDG-PET-CT	Visual	Partial volumes effects (PVE) maps for inspection of brain regions	Good agreement between the two modalities; hypoperfusion and hypometabolism were observed in areas including basifrontal, anterior temporal lobe, left posterior part of the temporal lobe, and left parietal lobe
Dolui et al. [2020] [30]	Case-control	Mild cognitive impairment (MCI)	50/35	Pseudo continuous ASL-MRI /FDG-PET-CT	Quantitative	t-score maps	ASL-MRI and FDG-PET have demonstrated similar areas of abnormalities including the medial temporoparietal regions
						Receiver-operator characteristic curve (ROC-curve) analysis	Similar area under the curve (AUC) between ASL and FDG
Fällmar et al. [2017] [20]	Case-control	Frontotemporal dementia (FTD)	20/38	Pseudo continuous ASL-MRI /FDG-PET	Visual	Sensitivity and specificity	ASL-MRI had higher specificity (0.84), but significantly lower sensitivity (0.53 vs. 0.96) than FDG-PET
		Alzheimer’s disease (AD)	25/38				
Musiek et al. [2012] [31]	Case-control	Alzheimer’s disease (AD)	15/19 (qualitative assessment)	Pseudo continuous ASL-MRI /FDG-PET	Visual	Receiver-Operator curve (ROC) analysis, and sensitivity/specificity analysis	Readers were more confident with FDG-PET images than ASL-MRI ones; both modalities displayed comparable sensitivity and specificity measures
			13/18 (quantitative assessment)		Quantitative		Both modalities demonstrated excellent diagnostic accuracy, with FDG-PET having AUC of 0.9 and that of ASL-MRI being 0.91
Nedelska et al. [2018] [32]	Case-control	Dementia with Lewey bodies (DLB)	19/76	Pseudo continuous ASL-MRI /FDG-PET and tau-PET	Quantitative	Quantitative maps, like voxel-wise ones, derived from Statistical Parametric Mapping	Hypometabolism and hypoperfusion patterns of the cortex showed a clear similarity among DLB patients in areas including precuneus, cuneus and posterior parieto-occipital cortices
		Alzheimer’s disease (AD)	19/76			ROC-curve analysis	FDG-PET performed better with AUC of 0.91, but ASL-MRI also had good accuracy with AUC of 0.8
Riederer et al. [2018] [33]	Case-control	Mild cognitive impairment (MCI)	20/11	Pulsed ASL-MRI /FDG-PET	Quantitative	Voxel-wise analyses of variance, volume of interest (VOI), and independent component analyses	For MCI patients, FDG-PET revealed hypometabolism patterns in the precuneus unlike ASL-MRI
		Alzheimer’s disease (AD)	45/11				For AD patients, both ASL-MRI and FDG-PET showed uniform patterns of hypoperfusion and hypometabolism respectively in areas including precuneus, parietal, temporal, and occipital cortex
Tosun & Jagust et al. [2016] [34]	Case-control	Early mild cognitive impairment (early-MCI)	30/34	ASL-MRI /FDG-PET	Quantitative	Partial least squares (PLS) method generated maps	Differences in the whole-brain perfusion and metabolism patterns
		Late mild cognitive impairment (late-MCI)	25/34			Sensitivity and specificity measures	Insignificant differences in sensitivity and specificity; however, FDG-PET had shown better measures in diagnosing AD and late MCI
		Alzheimer’s disease (AD)	20/34				
Tosun & Rabinovici et al. [2016] [35]	Case-control	Behavioral variant of frontotemporal dementia (bvFTD)	32/15	ASL-MRI /FDG-PET	Quantitative	Partial least squares (PLS) method generated maps	Spatial regions differentiating each disorder as derived from ASL-MRI and FDG-PET are similar
		Alzheimer’s disease (AD)	28/15			Sensitivity and specificity	Sensitivity and specificity measures of ASL and FDG-PET were uniform in discriminating AD, bvFTD, and control subjects. In differentiating AD from control participants, sensitivity and specificity of ASL was 0.86 and 0.92, whereas that of FDG-PET was 0.78 and 1 respectively
Verclytte et al. [2016] [25]	Prospective	Early-onset Alzheimer’s disease (EOAD)	37/0	Pseudo continuous ASL-MRI /FDG-PET-CT	Quantitative	P-maps	Hypometabolic regions were more extensive than hypoperfused ones. ASL maps highlighted changes in frontal lobes unlike FDG-PET
Verfaillie et al. [2015] [36]	Case-control	Frontotemporal dementia (FTD)	12/10	Pseudo continuous ASL-MRI /FDG-PET	Quantitative	Voxel-wise comparison Region-of-interest (ROI) and correlation	Perfusion and metabolism maps showed significant decrease in signal for AD patients in both precuneus and inferior parietal lobule As for both AD and FTD, there was a decrease in metabolism and perfusion in medial prefrontal cortex
		Alzheimer’s disease (AD)	18/10			ROC curve analyses	Area under curve were somehow comparable between FDG-PET and ASL-MRI pertaining the precuneus (0.74 versus 0.72) and inferior parietal lobule (0.94 versus 0.85), but not the medial prefrontal cortex region (both 0.68)
Weyts et al. [2017] [26]	Retrospective	Different types of dementia	9 demented patients/0	Pseudo continuous ASL-MRI /FDG-PET-CT	Visual	Inter- and intramodality agreement	Inter and intramodality agreements were insignificantly different among both modalities
						Regional agreement	Between modalities, intermodality agreement was similar in some brain regions including the precuneus
						Diagnostic accuracy	For ASL-MRI diagnostic accuracy is 5/9, and that of FDG-ASL is 7/9

To execute the meta-analysis, studies which have reported sensitivity and specificity measures of diagnostic accuracy were combined and summarized. Six out of the fourteen total articles included for the systematic review have clearly reported sensitivity and specificity values. However, there were more than one report of sensitivity/specificity for some studies based on either the dementia-related disease investigated (some studies have reported two or more diseases), or the method of assessing data (visual versus quantitative).

As it is demonstrated in Table 2, each study of the six articles included in the meta-analysis has documented one or more sets of sensitivity and specificity measures of diagnosis. Positive and negative predictive values were derived and calculated from the data available in the given articles.

**Table 2:** Meta-analysis study characteristics

Citation	Target Condition	Sample Size (Diseased/ Healthy)	Assessment Method (Visual/Quantitative)	Sensitivity (FDG-PET/ASL-MRI)	Specificity (FDG-PET/ASL-MRI)	Positive predictive value (FDG-PET/ASL-MRI)	Negative predictive value (FDG-PET/ASL-MRI)
Anazodo et al. [2017] [27]	Frontotemporal dementia (FTD)	10/10	Visual	0.88/0.66	0.90/0.62	0.90/0.63	0.88/0.65
Ceccarini et al. [2020] [19]	Different types of dementia	27/30	Visual	0.93/0.64	0.70/0.71	0.73/0.66	0.91/0.68
			Quantitative	0.79/0.57	0.63/0.81	0.65/0.72	0.76/0.67
Fällmar et al. [2017] [20]	Frontotemporal dementia (FTD)	20/38	Visual	0.96/0.53	0.54/0.84	0.71/0.80	0.91/0.60
	Alzheimer’s disease (AD)	25/38					
Musiek et al. [2012] [31]	Alzheimer’s disease (AD)	15/19 (qualitative assessment)	Visual	0.66/0.63	0.97/0.92	0.95/0.86	0.78/0.76
		13/18 (quantitative assessment)	Quantitative	0.72/0.77	0.92/0.92	0.87/0.87	0.82/0.85
Tosun & Jagust et al. [2016] [34]	Early mild cognitive impairment (early-MCI)	30/34	Quantitative	0.90/0.83	0.88/0.84	0.86/0.82	0.90/0.84
	Late mild cognitive impairment (late-MCI)	25/34		0.87/0.74	0.88/0.74	0.84/0.67	0.90/0.79
	Alzheimer’s disease (AD)	20/34		0.95/0.80	0.94/0.88	0.90/0.79	0.96/0.88
Tosun & Rabinovici et al. [2016] [35]	Behavioral variant of frontotemporal dementia (bvFTD)	32/15	Quantitative	0.79/0.78	1.00/0.92	1.00/0.96	0.62/0.59
	Alzheimer’s disease (AD)	28/15		0.78/0.86	1.00/0.92	1.00/0.95	0.70/0.77

### Quality assessment

**Fig. 2:**
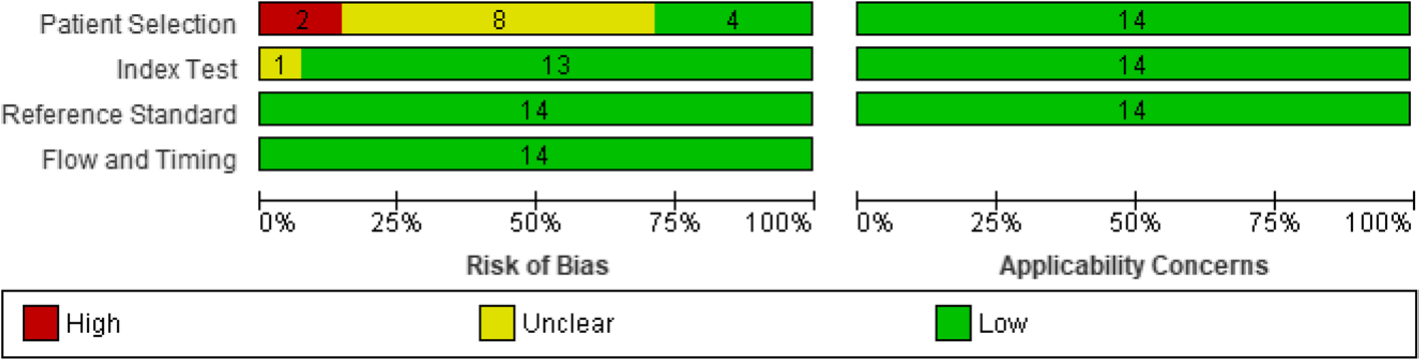
Risk of bias and applicability concerns graph: review authors’ judgements about each domain presented as percentages across included studies

**Fig. 3:**
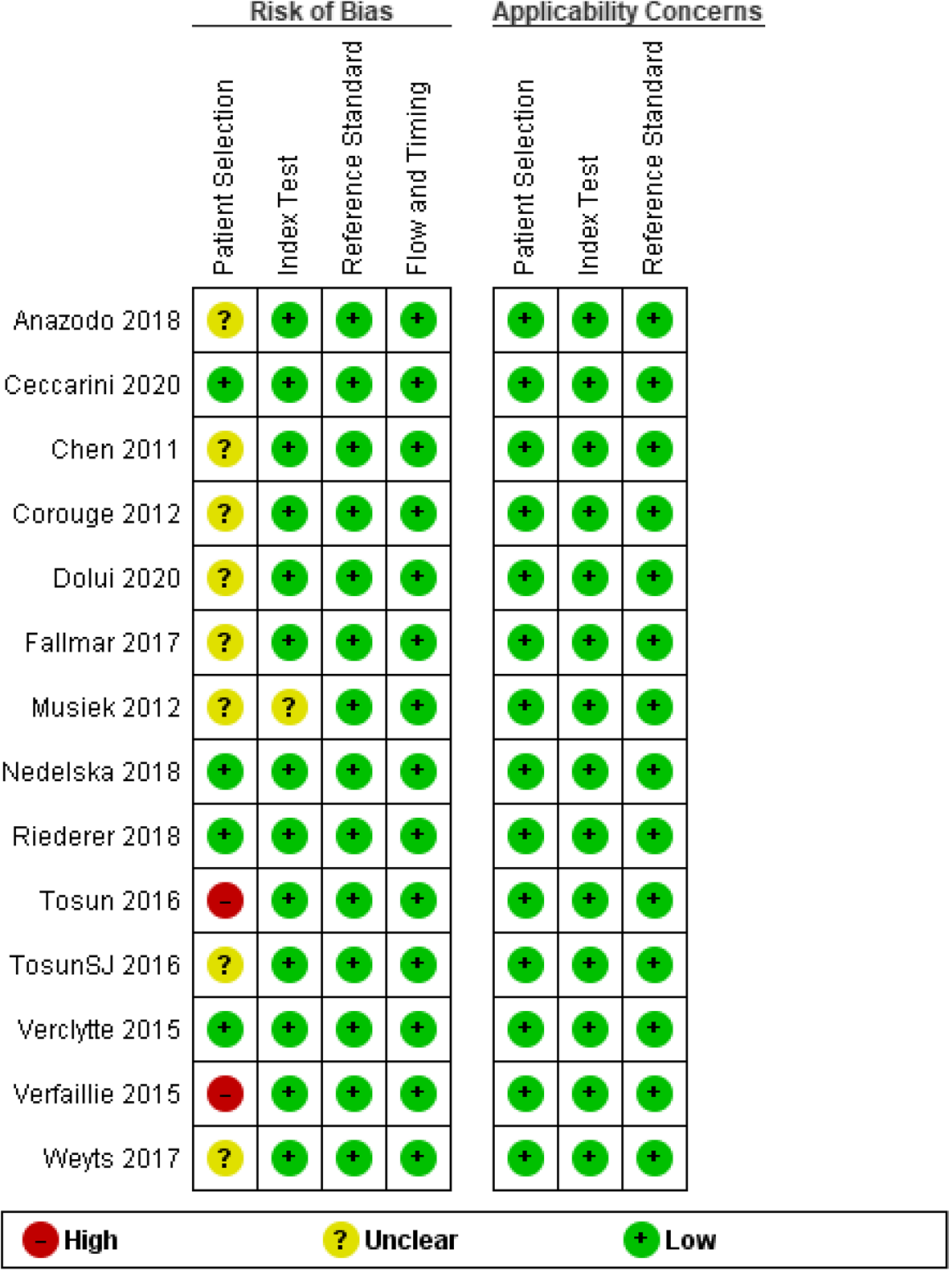
Risk of bias and applicability concerns summary: review authors’ judgements about each domain for each included study

Risk of bias was significantly high in 2 out of the 14 articles in the patient selection domain, whereas 8 articles had unclear risk of bias, and 4 articles showed low risk. In the index test domain, only one study showed unclear risk, but the rest of the articles had low risk of bias. All articles had low risk in the remaining domains along with the applicability concerns (Fig. 2). Figure 3 further illustrates the risk of bias and applicability concerns summary per article.

### Qualitative analysis

#### AD

Several studies found similar perfusion and metabolism brain maps when assessing patients with Alzheimer’s disease. Hypometabolism and hypoperfusion patterns within these studies coincided in regions including the bilateral angular gyri and posterior cingulate [28], precuneus, parietal, temporal, and occipital cortices [31, 33], and inferior parietal lobule [35, 36]. On the contrary to these findings, the other set of the studies exhibited significant differences between both imaging techniques. Fällmar et al. compared the diagnostic value of both modalities in evaluating AD. Lower sensitivity was registered for ASL-MRI (0.53) versus that of FDG-PET (0.96). It indicated that hypometabolic areas indicating abnormality were sparser than those hypoperfused ones [20]. Likewise, Tosun et al. and Verclytte et al. deduced that whole-brain metabolism and perfusion patterns were significantly different [25, 34].

#### FTD and DLB

Other studies investigated frontotemporal dementia by which some of them illustrated similar patterns of hypoperfusion and hypometabolism in the medial and lateral fronto-orbital, inferior, middle, superior frontal, precuneus, insular, and medial prefrontal cortices [35, 36]. On the other side, Anazodo et al. found that hypometabolism areas exceeded that of hypoperfused ones [27]. Comparably, Fällmar et al. showed that ASL-MRI has lower sensitivity, indicating that areas of hypometabolism are far more spread than those of produced by ASL-MRI [20].

#### MCI

Dolui et al. studied the diagnostic value of ASL-MRI and FDG-PET in evaluating patients with mild cognitive impairment on the AD continuum. Brain maps showed abnormalities in common areas including the medial temporoparietal regions [30]. In contrary to what preceded, results from both Riederer et al. and Tosun et al. suggested no overlapping metabolism and perfusion areas among a sample of patients with MCI [33, 34].

#### Others

Corouge et al. examined semantic dementia. Hypoperfusion and hypometabolism were observed in areas including basifrontal, anterior temporal lobe, left posterior part of the temporal lobe, and left parietal lobe [29]. As for dementia with Lewy bodies, Nedelska et al. results indicated a similarity in the hypometabolism and hypoperfusion brain patterns of the cortex in areas including precuneus, cuneus and posterior parieto-occipital cortices [32].

Weyts et al. probed several dementia-related diseases. Intermodality agreement was similar in some brain regions including the precuneus, anterior cingulate, anterior temporal lobe, and primary sensorimotor area [26]. Conversely to Weyts, ceccarini et al. results displayed, after examination of multiple dementia-related diseases, that FDG-PET manifested more volume and intensity abnormalities than ASL-MRI [19].

### Meta-analysis

A meta-analysis was employed to summarize the sensitivity and specificity measures of diagnostic accuracy while comparing both imaging techniques. Table 2 summarizes all extracted and derived data from the six articles included in the meta-analysis.

#### Forest plots

**Fig. 4:**
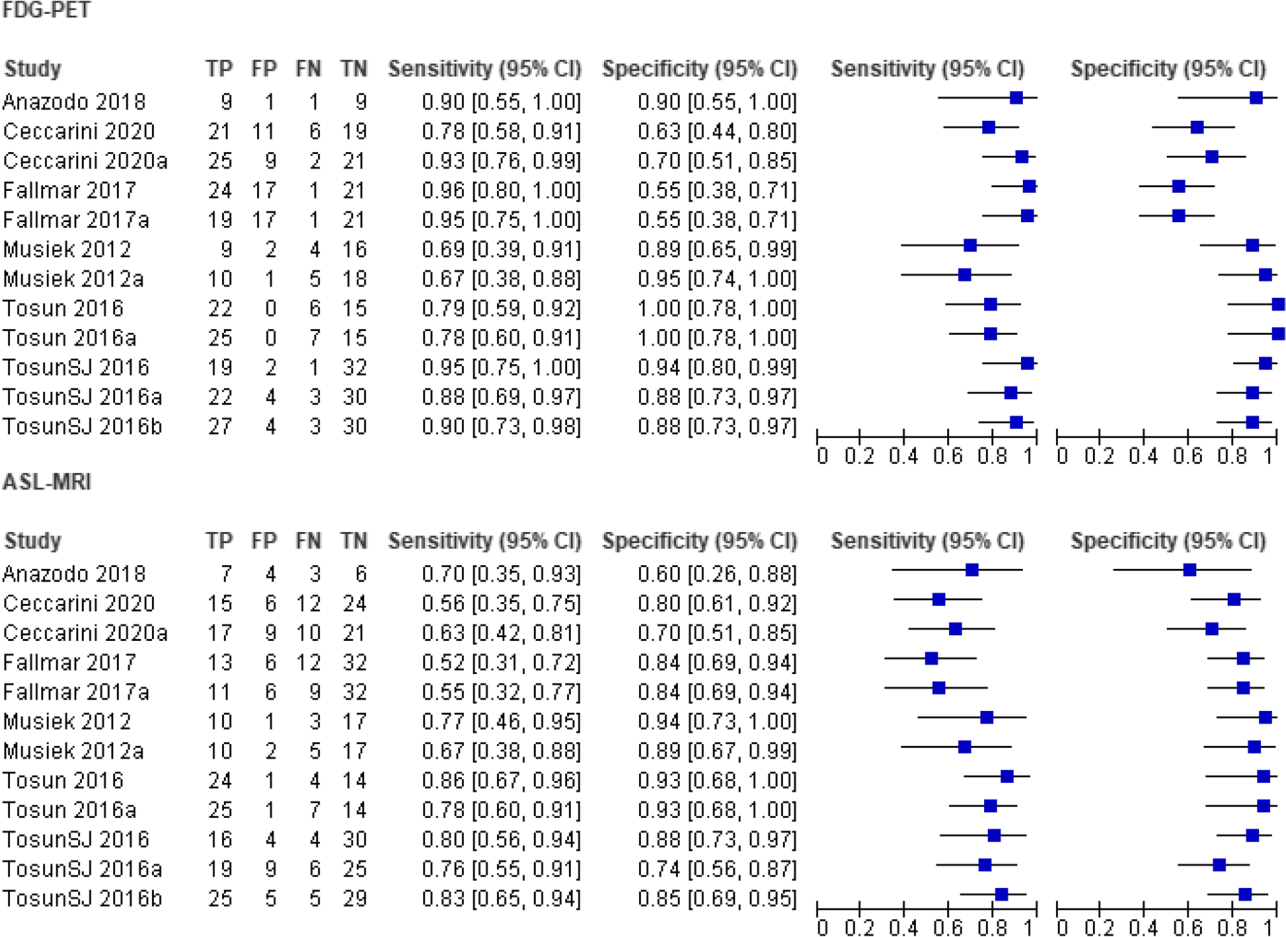
Forest plot of tests: 1 FDG-PET, 2 ASL-MRI.

Forest plots were created to summarize the diagnostic accuracy measures of FDG-PET and ASL-MRI. As it is clarified in Fig. 4, twelve total reports of sensitivity/specificity measures were employed from the six articles included. Articles which had more than one set of sensitivity/specificity measures were registered as a copy to the original study reference, with a letter (a) suggesting a first copy, and a letter (b) suggesting a second copy. It can be noticed from the plot that each report is defined by the dementia and analysis type.

#### Pooled sensitivity and specificity

From the twelve reported sets of sensitivity and specificity measures of diagnostic accuracy, the mean value has been calculated. Concerning sensitivity, the estimated pooled value for FDG-PET is 0.858, much greater than that of ASL-MRI (0.71) (Table 3). On the hand, estimated pooled specificity of FDG-PET and ASL-MRI are quite similar, with values being 0.863 and 0.834 respectively (Table 3).

**Table 3:** Pooled sensitivity and specificity measures of FDG-PET and ASL-MRI (lower and upper confidence intervals)

	Imaging Technique					
	FDG-PET		ASL-MRI			
**Parameter**	**Estimate**	**2.5% CI**	**97.5% CI**	**Estimate**	**2.5% CI**	**97.5% CI**
**Sensitivity**	0.858	0.800	0.902	0.71	0.635	0.775
**Specificity**	0.863	0.745	0.931	0.834	0.781	0.877
**False Positive Rate**	0.137	0.069	0.255	0.166	0.123	0.219

#### Summary-ROC curve analysis

**Fig. 5:**
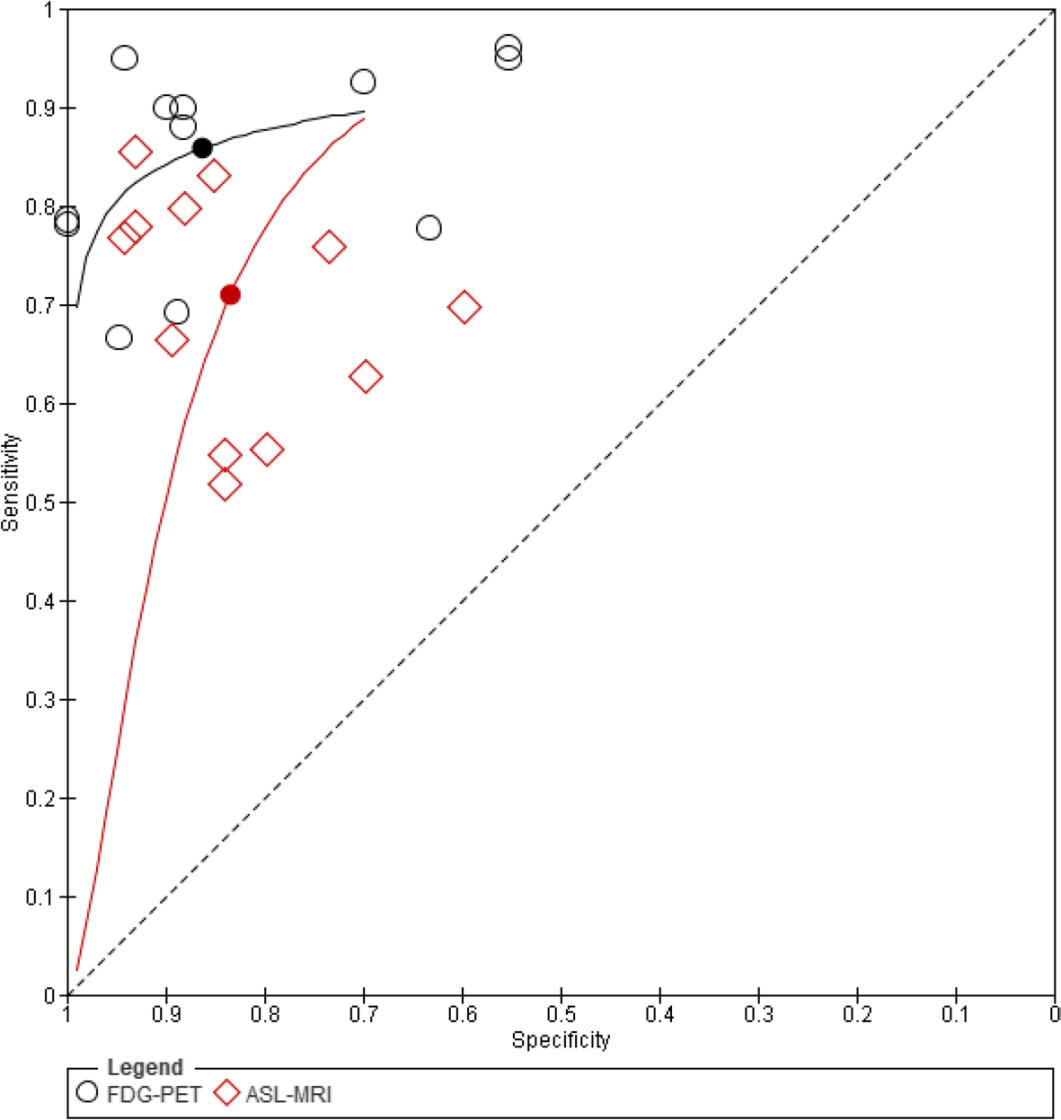
Summary ROC Plot of tests: BLACK: FDG-PET, RED: ASL-MRI.

A ROC-curve was created to further illustrate the results of the meta-analysis conducted as seen in Fig. 5. The curve was constructed based on a bivariate model using parameters calculated using the MetaDTA software [24]. It can be noted from figure five that FDG-PET demonstrated a higher overall diagnostic performance than ASL-MRI. Summary point of FDG-PET is higher than that of ASL-MRI. In addition, area under curve (AUC) was estimated using RStudio software per imaging modality. The SROC curve describing FDG-PET has an AUC of 86.7%, greater than that of ASL-MRI being 84.2%.

#### Heterogeneity considerations

To investigate the heterogeneity of the studies included in the meta-analysis and understand whether there are factors affecting the variability of both diagnostic measures, sensitivity and specificity, I2 index based on the approach described by Zhou and Dendukuri [37] was extracted from mada package on RStudio. The later approach is used for bivariate meta-analysis, and it therefore considers the correlation between sensitivity and specificity measures. It offers a better explanation for the variation within diagnostic test accuracy studies than the model derived by Higgins and Thompson [38].

The heterogeneity I2 index, as described by Zhou and Dendukuri [37], equals to 0% when taking into consideration FDG-PET studies, and it is 6% when considering ASL-MRI studies separately. Both percentages indicate that the heterogeneity of studies included is exceptionally low for both modalities, implying that there isn’t a significant variation between and within studies.

## Discussion

Dementia comes in many forms, and it is portrayed through various diseases including Alzheimer’s disease, fronto-temporal dementia, and dementia with Lewy bodies. FDG-PET is a commonly used imaging modality in the diagnosis of the varying dementia-related diseases. However, FDG-PET is known for being expensive and selectively available. Thus, seeking an alternative to FDG-PET is a common target for researchers and clinicians. ASL-MRI is a technique navigated by MRI. It is far cheaper than FDG-PET and is rather widely secured for people. Thereby, the following systematic review and meta-analysis was performed to compare literature findings related to the diagnostic ability of FDG-PET and ASL-MRI, and to conclude whether ASL-MRI is an eligible efficient alternative for FDG-PET.

A search of the available electronic literature review yielded a total of fourteen articles to be reviewed and assessed. All articles were qualitatively evaluated and systematically reviewed. Nonetheless, a meta-analysis was performed combining six out of the fourteen articles, for they reported sensitivity and specificity measures of diagnostic accuracy. Our results have shown, from a qualitative perspective, that studies are on opposite sides of the spectrum. Some studies have identified metabolism and perfusion brain maps to overlap in various brain regions depending on the disease investigated. However, other studies couldn’t find overridden brain regions among metabolism and perfusion brain maps, emphasizing that FDG-PET performed better at the diagnostic level. From a quantitative perspective, our meta-analysis revealed, as per the SROC-curve and AUC measures, that FDG-PET with an AUC of 86.7%, displays a better diagnostic performance than ASL-MRI with an AUC of 84.2%. The pooled sensitivity of FDG-PET is 0.858, significantly higher than that of ASL-MRI (0.71). Specificity was rather similar among both techniques (0.863 (FDG-PET) versus 0.834 (ASL-MRI)).

Generally speaking, our results indicate that FDG-PET still holds an advantage over ASL-MRI in diagnosing dementia. This could be explained by the fact that FDG-PET measures glucose metabolism which is highly sensitive to neuronal and synaptic activity changes reflecting back directly any degeneration or alteration [39]. On the other side, ASL-MRI measures cerebral blood flow which is coupled to neuronal activity; activation in certain brain regions would normally increases the blood flow to these regions [40]. However, ASL-MRI is characterized with low temporal resolution. Thus, changes with high pace are often not detected or lagged behind [41]. Nonetheless, studies included for the following review differed on various aspects including the nature of the disease investigated (early-onset versus late-onset), the sample size provided per each case, and the imaging techniques involved. Such discrepancies could have introduced bias within the deduced results.

Our findings somewhat coincide with a previous review comparing the diagnostic performance of ASL-MRI to FDG-PET and other neuroimaging modalities in the diagnosis of various neurological diseases including dementia. Quantitative summary of results derived from literature have indicated that there’s a regional overlap between hypometabolism and hypoperfusion data when considering patients suffering from dementia like AD and FTD patients [42]. The latter conclusion calls for investigating the possibility of securing an alternative to FDG-PET.

The given systematic review and meta-analysis is the first review to summarize the findings comparing the diagnostic accuracy of FDG-PET and ASL-MRI in solely differentiating demented patients from healthy ones. In addition, qualitative and quantitative analyses were both employed. Using qualitative analysis, a summary of the results extracted from studies was outlaid. Quantitatively, a meta-analysis of sensitivity and specificity measures of diagnostic accuracy was undertaken to compare the diagnostic value of both modalities.

Despite the significance of this systematic review, it still holds several shortcomings to be highlighted. The number of articles included is low, and the type of our review is a diagnostic test accuracy (DTA) review. This type of reviews is known to be characterized with several limitations. The majority of studies included for the review followed a case-control design, and such design might exaggerate the diagnostic value of tests [43]. Moreover, this type of review doesn’t always provide a clear explanation of the selection criteria and sampling of participants [44].

Heterogeneity pertaining results variation is another limitation in a diagnostic accuracy test review. As different studies have used different cut-off values in determining the sensitivity and specificity measures of diagnostic accuracy, results recorded would substantially vary from one study to another. However, our meta-analysis revealed a low heterogeneity index, which could be attributed to having several sets of sensitivity and specificity in the same study, decreasing the variations across studies, which is counted as a great advantage to the systematic review.

With the overall results suggesting that FDG-PET performs better at the diagnostic level for distinguishing dementia patients, still an undeniable part of the results shows that ASL-MRI somewhat performs in a similar way to FDG-PET. Qualitative findings did mention that metabolism and perfusion brain maps override each other in specific regions. In addition, while comparing the diagnostic accuracy between FDG-PET and ASL-MRI, specificity was somehow close in value which indicates a similar performance at that level. This definitely hints on the possibility that ASL-MRI could replace FDG-PET for the many advantages it possesses, if researchers can prove that this technique can guarantee an undistinguishable sensitivity from that of FDG-PET. Thus, it is recommended for future research to explore the effect of some variations, like the disease investigated or the method of assessing the imaging data, on the studies’ heterogeneity in order to decrease the impact of the heterogeneity-evoking factors, and to settle for more accurate comparisons between the two imaging modalities.

## Conclusion

In spite of the former limitations, our systematic review has successfully met the main research objective. With the systematic review and meta-analysis undertaken, a well-established comparison between FDG-PET and ASL-MRI in diagnosing dementia patients was maintained. While identifying any overlap in metabolism and perfusion brain regions, qualitative findings showed either a similar diagnostic ability of ASL-MRI to FDG-PET, or a diagnostic advantage of FDG-PET over ASL-MRI. On the other hand, the meta-analysis implemented, which summarized sensitivity and specificity measures of diagnostic accuracy, revealed a higher performance associated with FDG-PET. Henceforth, our results although favor FDG-PET in the diagnosis of dementia, still some evidence shedding light on an equivalent performance by ASL-MRI can certainly be further investigated.

## Data Availability

All data generated or analyzed during this study are included in this published article.

## Abbreviations

WHO: World health organization
AD: Alzheimer’s disease
VD: Vascular dementia
DLB: Dementia with Lewy bodies
CT: Computed tomography
PET: Positron emission tomography
fMRI: Functional magnetic resonance imaging
ASL-MRI: Arterial spin labeling - magnetic resonance imaging
FDG-PET: Fluorodeoxyglucose-positron emission tomography
PRISMA-DTA: Preferred Reporting Items for Systematic Reviews and Meta-Analyses- Diagnostic Test Accuracy
QUADAS-2: Quality Assessment of Diagnostic Accuracy Studies tool, version 2
TP: True positive
TN: True negative
FP: False negative
SROC-curve: Summary receiver-operator characteristic curve
REVMAN: Review manager
FTD: Frontotemporal dementia
SD: Semantic dementia
MCI: Mild cognitive impairment
bvFTD: Behavioral variant of frontotemporal dementia
EOAD: Early-onset Alzheimer’s disease
AUC: Area under curve

## References

[1] Gustafson L (1996). What is Dementia?. Acta Neurol Scand.

[2] Dening T, Sandilyan MB (2015). Dementia: definitions and types. Nurs Standard (2014+).

[3] World Health Organization. The ICD-10 classification of mental and behavioral disorders: clinical descriptions and diagnostic guidelines. World Health Organization; 1992.

[4] van der Flier WM, Scheltens P (2005). Epidemiology, and risk factors of Dementia. J Neurol Neurosurg Psychiatry.

[5] Alzheimer’s Disease International. World Alzheimer Report 2014: Dementia and Risk Reduction. (2014). www.alz.co.uk/research/world-report2014.

[6] Alzheimer’s Disease International. Dementia Statistics. (2013). www.alz.co.uk/research/statistics.

[7] Qiu C, Kivipelto M, Von Strauss E. Epidemiology of Alzheimer’s disease: occurrence, determinants, and strategies toward intervention. Dialogues Clin Neurosci. 2009;11(2):111–28. 10.31887/DCNS.2009.11.2/cqiu. 10.31887/DCNS.2009.11.2/cqiu PMC3181909 19585947

[8] Steinberg M, Shao H, Zandi P, Lyketsos CG, Welsh-Bohmer KA, Norton MC, Breitner JC, Steffens DC, Tschanz JT (2008). Point and 5‐year period prevalence of neuropsychiatric symptoms in Dementia: the Cache County study. Int J Geriatric Psychiatry: J Psychiatry late life Allied Sci.

[9] Jackson K, Barisone GA, Diaz E, Jin LW, DeCarli C, Despa F (2013). Amylin deposition in the brain: a second amyloid in Alzheimer Disease?. Ann Neurol.

[10] Matsui Y, Tanizaki Y, Arima H, Yonemoto K, Doi Y, Ninomiya T, Sasaki K, Iida M, Iwaki T, Kanba S, Kiyohara Y (2009). Incidence and survival of Dementia in a general population of Japanese elderly: the Hisayama study. J Neurol Neurosurg Psychiatry.

[11] Diagnostic criteria for dementia. (2019). https://www.dementia.org.au/files/helpsheets/Helpsheet-DementiaQandA11-DiagnosticCriteriaForDementia_english.pdf.

[12] Dubois B, Hampel H, Feldman HH, Scheltens P, Aisen P, Andrieu S, Bakardjian H, Benali H, Bertram L, Blennow K, Broich K (2016). Preclinical Alzheimer’s Disease: definition, natural history, and diagnostic criteria. Alzheimer’s Dement.

[13] Dipanjan B, Abilash M, Rub HM, Haider MB (2020). Neuroimaging in Dementia: a brief review. Cureus..

[14] Sarikaya I, Sarikaya A, Elgazzar AH (2018). Current status of 18F-FDG PET brain imaging in patients with Dementia. J Nucl Med Technol.

[15] Brown RK, Bohnen NI, Wong KK, Minoshima S, Frey KA (2014). Brain PET in suspected Dementia: patterns of altered FDG metabolism. Radiographics: A Review Publication of the Radiological Society of North America Inc.

[16] Sone D, Maikusa N, Sato N, Kimura Y, Ota M, Matsuda H (2019). Similar and differing distributions between 18F-FDG-PET and arterial spin labeling imaging in temporal lobe Epilepsy. Front Neurol.

[17] Detre JA, Leigh JS, Williams DS, Koretsky AP (1992). Perfusion imaging. Magn Reson Med.

[18] Raichle ME (1998). Behind the scenes of functional brain imaging: a historical and physiological perspective. Proc Natl Acad Sci.

[19] Ceccarini J, Bourgeois S, Van Weehaeghe D, Goffin K, Vandenberghe R, Vandenbulcke M, Sunaert S, Van Laere K (2020). Direct prospective comparison of 18F-FDG PET and arterial spin labelling MR using simultaneous PET/MR in patients referred for diagnosis of Dementia. Eur J Nucl Med Mol Imaging.

[20] Fällmar D, Haller S, Lilja J, Danfors T, Kilander L, Tolboom N, Egger K, Kellner E, Croon PM, Verfaillie SC, van Berckel BN (2017). Arterial spin labeling-based Z-maps have high specificity and positive predictive value for neurodegenerative Dementia compared to FDG-PET. Eur Radiol.

[21] McInnes MD, Moher D, Thombs BD, McGrath TA, Bossuyt PM, Clifford T, Cohen JF, Deeks JJ, Gatsonis C, Hooft L, Hunt HA (2018). Preferred reporting items for a systematic review and meta-analysis of diagnostic test accuracy studies: the PRISMA-DTA statement. JAMA.

[22] Whiting PF, Rutjes AW, Westwood ME, Mallett S, Deeks JJ, Reitsma JB, Leeflang MM, Sterne JA, Bossuyt PM (2011). QUADAS-2 Group*. QUADAS-2: a revised tool for the quality assessment of diagnostic accuracy studies. Ann Intern Med.

[23] Reitsma JB, Glas AS, Rutjes AW, Scholten RJ, Bossuyt PM, Zwinderman AH (2005). Bivariate analysis of sensitivity and specificity produces informative summary measures in diagnostic reviews. J Clin Epidemiol.

[24] Freeman SC, Kerby CR, Patel A, Cooper NJ, Quinn T, Sutton AJ (2019). Development of an interactive web-based tool to conduct and interrogate meta-analysis of diagnostic test accuracy studies: MetaDTA. BMC Med Res Methodol.

[25] Verclytte S, Lopes R, Lenfant P, Rollin A, Semah F, Leclerc X, Pasquier F, Delmaire C (2016). Cerebral hypoperfusion and hypometabolism detected by arterial spin labeling MRI and FDG-PET in early‐onset Alzheimer’s Disease. J Neuroimaging.

[26] Weyts K, Vernooij M, Steketee R, Valkema R, Smits M (2017). Qualitative agreement and diagnostic performance of arterial spin labelling MRI and FDG PET-CT in suspected early-stage Dementia: comparison of arterial spin labelling MRI and FDG PET-CT in suspected Dementia. Clin Imaging.

[27] Anazodo UC, Finger E, Kwan BY, Pavlosky W, Warrington JC, Günther M, Prato FS, Thiessen JD, Lawrence KS (2018). Using simultaneous PET/MRI to compare the accuracy of diagnosing frontotemporal Dementia by arterial spin labelling MRI and FDG-PET. NeuroImage. Clinical.

[28] Chen Y, Wolk DA, Reddin JS, Korczykowski M, Martinez PM, Musiek ES, Newberg AB, Julin P, Arnold SE, Greenberg JH, Detre J (2011). Voxel-level comparison of arterial spin-labeled perfusion MRI and FDG-PET in Alzheimer Disease. Neurology.

[29] Corouge I, Esquevin A, Le Jeune F, Ferré JC, Bannier E, Merck C, Belliard S, Barillot C, Gauvrit JY. Arterial Spin Labeling at 3T in semantic dementia: perfusion abnormalities detection and comparison with FDG-PET. InMICCAI 2012 Workshop on Novel Biomarkers for Alzheimer’s Disease and Related Disorders; 2012. p. 32–40. inserm-00730431, version 1.

[30] Dolui S, Li Z, Nasrallah IM, Detre JA, Wolk DA (2020). Arterial spin labeling versus 18F-FDG-PET to identify mild cognitive impairment. NeuroImage: Clin.

[31] Musiek ES, Chen Y, Korczykowski M, Saboury B, Martinez PM, Reddin JS, Alavi A, Kimberg DY, Wolk DA, Julin P, Newberg AB (2012). Direct comparison of fluorodeoxyglucose positron emission tomography and arterial spin labeling magnetic resonance imaging in Alzheimer’s Disease. Alzheimer’s Dement.

[32] Nedelska Z, Senjem ML, Przybelski SA, Lesnick TG, Lowe VJ, Boeve BF, Arani A, Vemuri P, Graff-Radford J, Ferman TJ, Jones DT, Nedelska Z, Senjem ML, Przybelski SA, Lesnick TG, Lowe VJ, Boeve BF, Arani A, Vemuri P, Graff-Radford J, Ferman TJ, Jones DT, Savica R, Knopman DS, Petersen RC, Jack CR, Kantarci K (2018). Regional cortical perfusion on arterial spin labeling MRI in dementia with Lewy bodies: Associations with clinical severity, glucose metabolism and tau PET. NeuroImage: Clinical..

[33] Riederer I, Bohn KP, Preibisch C, Wiedemann E, Zimmer C, Alexopoulos P, Förster S (2018). Alzheimer Disease and mild cognitive impairment: integrated pulsed arterial spin-labeling MRI and 18F-FDG PET. Radiology.

[34] Tosun D, Schuff N, Jagust W, Weiner MW (2016). Alzheimer’’s Disease Neuroimaging Initiative. Discriminative power of arterial spin labeling magnetic resonance imaging and 18F-fluorodeoxyglucose positron emission tomography changes for amyloid-β-positive subjects in the Alzheimer’s Disease continuum. Neurodegenerative Dis.

[35] Tosun D, Schuff N, Rabinovici GD, Ayakta N, Miller BL, Jagust W, Kramer J, Weiner MM, Rosen HJ (2016). Diagnostic utility of ASL-MRI and FDG-PET in the behavioral variant of FTD and AD. Annals of clinical and translational neurology..

[36] Verfaillie SC, Adriaanse SM, Binnewijzend MA, Benedictus MR, Ossenkoppele R, Wattjes MP, Pijnenburg YA, van der Flier WM, Lammertsma AA, Kuijer J, Boellaard R (2015). Cerebral perfusion and glucose metabolism in Alzheimer’s Disease and frontotemporal Dementia: two sides of the same coin?. Eur Radiol.

[37] Zhou Y, Dendukuri N (2014). Statistics for quantifying heterogeneity in univariate and bivariate meta-analyses of binary data: the case of meta‐analyses of diagnostic accuracy. Stat Med.

[38] Higgins JP, Thompson SG (2002). Quantifying heterogeneity in a meta-analysis. Stat Med.

[39] Mosconi L (2013). Glucose metabolism in normal aging and Alzheimer’s Disease: methodological and physiological considerations for PET studies. Clin Translational Imaging.

[40] Malpass K (2012). Arterial spin-labeled MRI for diagnosis and monitoring of AD. Nat Reviews Neurol.

[41] Borogovac A, Asllani I (2012). Arterial spin labeling (ASL) fMRI: advantages, theoretical constrains and experimental challenges in neurosciences. Int J Biomed Imaging.

[42] Zhang J (2016). How far is arterial spin labeling MRI from a clinical reality? Insights from arterial spin labeling comparative studies in Alzheimer’s Disease and other neurological disorders. J Magn Reson Imaging.

[43] Suttie M, Foroud T, Wetherill L, Jacobson JL, Molteno CD, Meintjes EM, Hoyme HE, Khaole N, Robinson LK, Riley EP, Jacobson SW (2013). Facial dysmorphism across the fetal alcohol spectrum. Pediatrics.

[44] Goh PK, Doyle LR, Glass L, Jones KL, Riley EP, Coles CD, Hoyme HE, Kable JA, May PA, Kalberg WO, Elizabeth RS (2016). A decision tree to identify children affected by prenatal alcohol exposure. J Pediatr.

